# Molecular Mechanisms Underlying Fungicide Resistance in Citrus Postharvest Green Mold

**DOI:** 10.3390/jof7090783

**Published:** 2021-09-21

**Authors:** Paloma Sánchez-Torres

**Affiliations:** Department of Food Biotechnology, Institute of Agrochemistry and Food Technology, Spanish National Research Council (IATA-CSIC), Calle Catedrático Agustín Escardino 7, Paterna, 46980 Valencia, Spain; psanchez@iata.csic.es; Tel.: +34-963-900-022

**Keywords:** citrus, fungicide resistance, postharvest, *Penicillium digitatum*, infection

## Abstract

The necrotrophic fungus *Penicillium digitatum* (Pd) is responsible for the green mold disease that occurs during postharvest of citrus and causes enormous economic losses around the world. Fungicides remain the main method used to control postharvest green mold in citrus fruit storage despite numerous occurrences of resistance to them. Hence, it is necessary to find new and more effective strategies to control this type of disease. This involves delving into the molecular mechanisms underlying the appearance of resistance to fungicides during the plant–pathogen interaction. Although mechanisms involved in resistance to fungicides have been studied for many years, there have now been great advances in the molecular aspects that drive fungicide resistance, which facilitates the design of new means to control green mold. A wide review allows the mechanisms underlying fungicide resistance in Pd to be unveiled, taking into account not only the chemical nature of the compounds and their target of action but also the general mechanism that could contribute to resistance to others compounds to generate what we call multidrug resistance (MDR) phenotypes. In this context, fungal transporters seem to play a relevant role, and their mode of action may be controlled along with other processes of interest, such as oxidative stress and fungal pathogenicity. Thus, the mechanisms for acquisition of resistance to fungicides seem to be part of a complex framework involving aspects of response to stress and processes of fungal virulence.

## 1. Introduction

Citrus fruits are important fruit crops around the world because they provide numerous nutrients that promote human health [1,2]. Citrus fruits are subject to different biotic or abiotic stresses during the postharvest period, which includes handling, shipping, storage, and marketing. In this context, fruit spoilage and food safety risks due to postharvest fungal diseases constitute some of the most significant threats [3]. *Penicillium digitatum* (Pers.:Fr) Sacc. (Pd), which causes green mold, is the major postharvest rot of citrus fruits [1]. Postharvest green mold causes huge economic losses worldwide every year and can account for up to 90% of the total postharvest damage to citrus fruits, especially in dry areas and subtropical climates [4,5].

**Figure 1:**
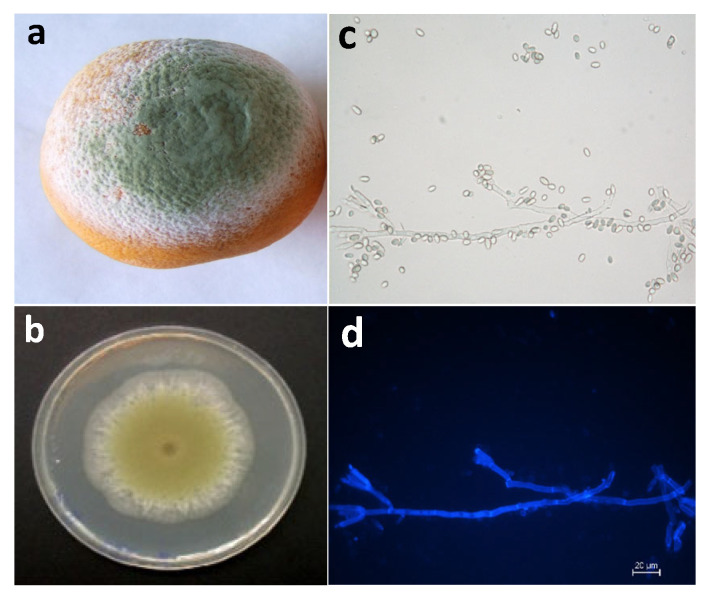
Green mold decay in orange fruit infected with *Penicillium digitatum*. (**a**) Orange fruit with green mold symptoms caused by *P. digitatum*; (**b**) typical aspect *P. digitatum* growing on PDA plates; (**c**) *P. digitatum* conidiphore with conidia borne terminally in chains observed by bright-field optical microscopy; (**d**) *P. digitatum* with fluorescence after staining with CFW.

Pd is capable of invading and infecting the fruit through wounds that are produced by environmental factors or during the harvest development, transport, and further treatments [6]. This fungus extends in oil glands through rind wounds [7], where they can access nutrients that promote germination of the conidia. The starting point of infection is the softer and more watery area on the surface of the skin where, with suitable temperature and optimal conditions, it progresses to give rise to a white mycelium that later turns to olive color due to the appearance of conidia [8,9] (Figure 1).

Pd whole genome sequencing has recently opened up new possibilities to investigate fungal factors related to host–pathogen interactions ranging from virulence factors to genes involved in fungicide resistance mechanisms. Understanding the infection process and the fungal strategy to overcome fungicides is one way to develop new forms of control [10,11].

The control of green mold is currently achieved through the application of synthetic compounds, such as imazalil, thiabendazole, pyrimethanil, and fludioxonil [12]. However, the continued use of chemicals to prevent fungal diseases has restricted their effectiveness and shelf life. The emergence of pathogenic fungi that is resistant to synthetic fungicides mainly used for the control of fungal infections poses a risk to the environment and consumer health [3], thus prompting research to develop new, more effective control tools.

## 2. Fungicide Resistance Has Become a Major Problem

Fungicides are crucial to preserve healthy, consistent, high-quality agricultural goods. Until 1970, almost all chemicals used to manage plant pathogens were multisite inhibitors that worked as protectors of diseases. In spite of their extensive use in some cases, resistance has not evolved to largely nonsystemic protectant fungicides due to their multisite modes of action [13]. However, since the introduction of site-specific fungicides in the late 1960s, fungicide resistance in plant pathogenic fungi has emerged as a major problem in crop control [14]. Since the 1970s, there has been an improvement in crop protection owing to systemic single-site fungicides that possess both protective and eradicating characteristics, such as methylbenzimidazole carbamates (MBC), sterol biosynthesis inhibitors (DMIs), external quinone inhibitors (QoI), and succinate dehydrogenase inhibitors (SDHI) (Table 1).

Resistance to fungicides results in reduction in sensitivity to certain compounds and is caused by an inherited adjustment of the fungus to that compound. It is normally due to either single or multiple genetic mutations. The identification of resistant isolates appears with a natural rate of genetic mutation, so the number of resistant strains is generally not affected by the application of a fungicide [15].

While fungicides effectively kill sensitive strains, resistant strains become dominant over time because pathogen populations are under selection pressure from continued fungicide use, leading to failure to control disease [16]. The fitness of the fungicide-resistant fungal isolates once selected will determine the permanence of the resistant genotypes. In some cases, it has been observed that resistant strains may have less aptitude than susceptible strains, thus requiring selection pressure of the fungicide to survive. Therefore, when the application of fungicides ceases, the number of resistant isolates in pathogen populations will decrease. On the other hand, the strong isolates present a fitness similar to the sensitive isolates, and they could remain for a long time even without the application of fungicides [17].

**Figure 2:**
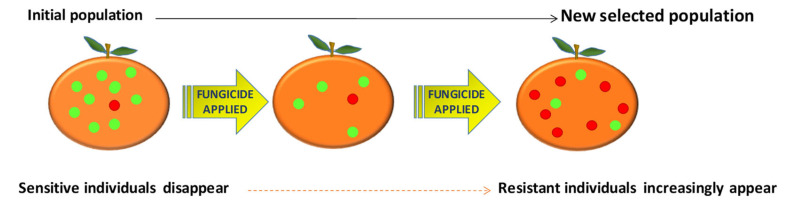
A diagram of the evolution of resistance to fungicides. This graphic shows an example of how selection pressure may take place. Initial population with little resistance evolves until resistance becomes widespread due to repeated fungicide applications. Adapted from Deising et al. [18].

Like in all organisms, there is genetic variation in pathogenic fungal populations. This variation provides the starting point from which fungicide resistance progresses. A complete population of resistant pathogenic fungi develops owing to natural selection, in which the environment favors the reproduction and proliferation of resistant forms. Individual fungicide applications can be considered the “selection events” that promote this process, selectively killing susceptible fungi. However, any resistant mutant will survive these events and subsequently have the opportunity to grow and reproduce without competition from susceptible fungi. After one application, this increasingly resistant population can proliferate and reproduce [18] (Figure 2).

**Figure 3:**
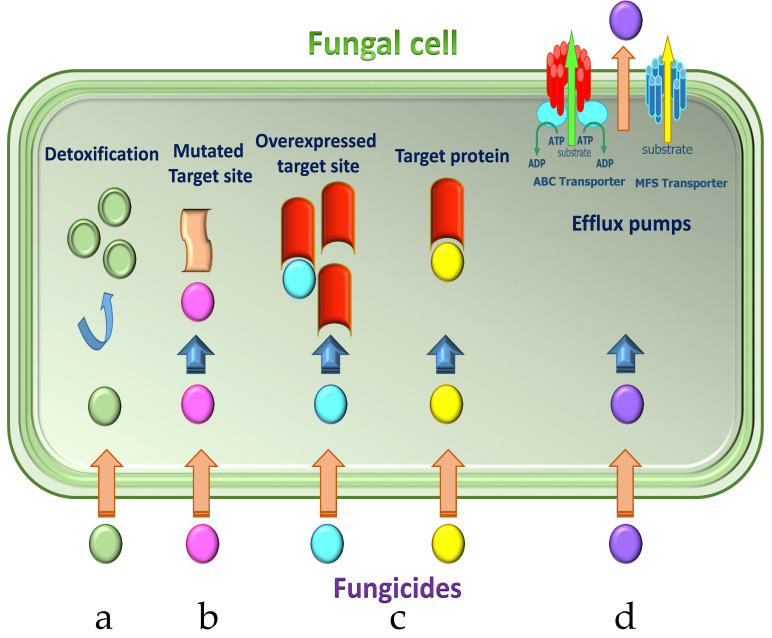
Main mechanisms of acquiring resistance to fungicides in *P. digitatum.* Mechanisms of resistance to single-site fungicides: (**a**) detoxification of fungicide through metabolic enzymes; (**b**) reduced fungicide binding due to alteration of the target protein; (**c**) overexpression of the target protein; (**d**) efflux pumps removing fungicide out of the cell. Adapted from Lucas et al. [17].

Resistance to fungicides might be due to various procedures [19,20,21,22], including (a) reduced fungicide binding due to alteration of the target site, (b) overexpression of the target protein, (c) reduced fungicide absorption due to efflux pump removing toxic compounds, and (d) metabolic degradation of the fungicide through detoxification (Figure 3).

The mechanisms involved in the appearance of resistance to fungicides in populations of field pathogens entail the study of the processes that intervene in the reduction of sensitivity to the compound and the genetic basis of the resistance trait. As there are several classes of single-site inhibitors, it is likely that there are several mechanisms that lead to fungicide resistance in plant pathogens, including the major citrus pathogen Pd.

The newest biotechnology for genome editing is a promising tool for the development of disease-resistant crops in the future [23]. Thus, investigating the molecular mechanisms underlying fungicide resistance in plant–pathogen interactions is essential for developing new and better approaches for efficiently controlling plant diseases.

The fruit–pathogen interaction is fundamental for the progression of fungal pathogen. It has therefore prompted great interest in the research community, and numerous studies have been undertaken in relation to the virulence of pathogens and the response of the fruit to infection [3]. In the citrus–Pd interaction, it has been advantageous to have the complete sequence of the Pd genome as well as the genetic transformation systems for Pd [5,8,24]. This has massively facilitated knowledge of the molecular processes underlying the pathogenicity of Pd [25].

This review presents an overall view of recent advances in the fungicide resistance mechanisms of postharvest citrus green mold, providing valuable information on the molecular procedures involved in the achievement of resistance to different chemicals, either to a single compound or to several compounds at the same time in the context of the fruit–pathogen interaction. This information is beneficial for developing novel and safer strategies to prevent postharvest green mold in citrus fruits and contributes substantially to knowledge on fungal disease management.

## 3. Molecular Mechanisms of Fungicide Resistance

Fungicide resistance can evolve differently based on the characteristics of the fungicide (fungicide class) (Table 1).

### 3.1. Methyl Benzimidazoles (MBCs)

The mechanism of benzimidazole-type fungicides, which includes thiabendazole (TBZ), involves binding to β-tubulins. This prevents the assembly of microtubules and cell division during mitosis and therefore results in toxicity to fungal cells [26,27].

Resistance to benzimidazole fungicides has been described in a wide variety of fungi. Frequently, the responsible mechanism corresponds to point mutations in the β-tubulin gene, which leads to the modification of some amino acids [28,29,30]. Among the numerous changes observed in the β-tubulin gene associated with resistance to MBC fungicides in phytopathogenic fungi, the most frequent have been in residues 198 and 200 [14]. It should be noted that the replacement of glutamic acid by alanine, valine, or glycine at position 198 and phenylalanine by tyrosine at position 200 can lead to varying levels of resistance to MBC fungicides [31,32]. In the particular case of TBZ, modifications in the TBZ binding site provides cellular resistance to it. Such variations usually occur at positions 198 or 200 of β-tubulin, although other changes are also possible [14,33]. In Pd, two different point mutations have been described as being responsible for TBZ resistance. Mutation at position 198 Glu to Lys was described by Ma and Michailides [14] based on studies performed in *Penicillium expansum* [27]. In Pd isolates found in citrus fruits from California packinghouses, resistance to TBZ was due to modification at position 200 of β-tubulin [34]. The same mutation was later described in Pd Spanish isolates collected from orchards and packinghouses [35], revealing that resistance mechanism is independent of fungicide pressure. Among Pd isolates collected from citrus in Taiwan, resistance to TBZ was associated with the most frequent β-tubulin mutations at codon 198 or 200 [36].

However, until now, no study has described the molecular process by which genetic variations in β-tubulin prevent the binding of fungicide. Recently, research carried out on *Podosphaera xanthii* using a combination of different approaches proposed that the MBC fungicide binding site in β-tubulin does not participate in the residues responsible for fungal resistance [37]. As a mechanism, it is suggested that when MBC fungicides spontaneously bind to β-tubulin in sensitive fungi, their conformation is altered and adequate polymerization in microtubules occurs; however, this does not take place in resistant strains, where there is a conformational change promoted by specific modifications.

### 3.2. Demethylation Inhbithors (DMIs)

DMI fungicides hamper the activity of the cytochrome P450-dependent sterol 14α-demethylase (Cyp51) and thus block C14-demethylation of lanosterol, a precursor of ergosterol in fungal pathogens [38]. DMIs encompass one of the most relevant groups of fungicides that prevent different plant diseases by inhibiting the activity of cytochrome P450-dependent sterol 14α-demethylase (P45014DM) and were first used in agriculture in the 1970s [39]. Imazalil is a demethylation inhibitor (DMI) that blocks ergosterol biosynthesis [40,41] and is frequently used to prevent postharvest diseases of citrus fruits worldwide due to its curative and antisporulant action against Pd [42]. *CYP51* encodes sterol 14α−demethylase, an enzyme responsible for ergosterol biosynthesis [43], and is the target of DMI fungicides.

The main mechanisms that provide DMI resistance are (i) modifications in *CYP51* or (ii) high expression of *CYP51.* Different procedures causing DMI resistance have been reported. They are mediated either by specific changes in the coding region [44,45,46] or by augmenting gene transcription due to an insertion in the promoter [47]. There are three homologues of the sterol 14α-demethylase-encoded CYP51 gene in Pd, namely PdCYP51A [48], PdCYP51B, and PdCYP51C [49]. The first mechanism involving modifications in CYP51 has been described in several pathogens. A single change, such as the substitution of a phenylalanine for a tyrosine at residue 136 (Y136F) of CYP51, led to resistance to DMI in *Uncinula necator* [50], *Erysiphe graminis f.sp. hordei* [51], *Erysiphe necator* [52], and *P. expansum* [44], while two single nucleotide changes were found to result in amino acid substitutions Y136F and K147Q in CYP51 in *Blumeria graminis* [53]. Other changes have been described in *Tapesia* sp. [54], *Penicillium italicum* [55], *Ustilago maydis* [56], *Blumeriella jaapii* [57], and *Mycosphaerella graminicola* [58]. In Pd, no *PdCYP51A* point mutations were found to be responsible for Pd resistance to IMZ or other DMI [35] or to prochloraz [46]. On the other hand, in *PdCYP51B*, no variations in the gene were initially detected in isolates resistant to IMZ [59]. However, recently, different substitutions of *PdCYP51B* have been found corresponding to different levels of sensitivity to prochloraz, namely Y136H and Q309H in high resistant strains, G459S and F506I in medium resistant strains, and Q309H in low resistance strains [46].

The other process responsible for resistance to DMI is change in the level of CYP51 transcription [60]. The most frequent mechanism is the presence of insertions in the promoter region in the phytopathogenic fungus, as was the case in *B. jaapii* [57], *Venturia inaequalis* [61], *Monilinia fructicola* [62], and *M. graminicola* [58]. This process has also been linked to the imazalil resistance in Pd. The first mechanism described was the presence of five tandem repeats of a 126 bp transcriptional enhancer in the promoter region of *PdCYP51A*, resulting in the overexpression of *PdCYP51A* [40]. These specific repeats allowed the design of a molecular tool to identify IMZ-resistant Pd. The method is based on the detection of the tandem repeat of a 126 bp sequence in the promoter region of *PdCYP51A* by PCR [48]. Furthermore, a new 199 bp sequence was identified that disrupts the 126 bp transcriptional enhancer, resulting in increased expression of *PdCYP51A* [63]. On the other hand, in a study carried out in 75 Spanish strains of Pd, resistance to DMIs in Pd did not correlate with the 126 bp tandem repeats of *PdCYP51A* [35]. Therefore, in the new CYP51 gene (*PdCYP51B*) identified in Pd, a unique insertion of 199 bp was observed in the promoter region that was associated with its overexpression and resistance to DMI fungicides [49]. The same insertion, but reduced to 195 bp, was identified in Spanish Pd isolates, demonstrating that overexpression of this gene is the predominant mechanism for resistance to DMI and in particular to IMZ [59]. This insert was identical to that described by Ghosoph et al. [63] in *PdCY51A*, which also conferred resistance to IMZ. Thus, the *PdCYP51B* enhancer actually behaves like a transposon that acts as the MITE element *PdMLE* [64] and is more stable and predominant than the *PdCYP51A* enhancer. In fact, when present in *PdCYP51B*, it is not compatible with the presence of the five tandem repeats of 126 bp enhancer of *PdCYP51A* [59].

### 3.3. Quinone Outside Inhibitors (QoI)

QoI fungicides impede respiration by binding to the Qo site of the cytochrome bc1 enzyme complex, resulting in energy deficiency and leading to the death of fungal pathogens [65]. This mode of action in QoI fungicides results in frequent appearance of QoI resistance in specific phytopathogenic fungi.

As with other external quinone inhibitor (QoI) fungicides, azoxystrobin is highly effective in preventing a wide variety of plant diseases [20,66], including citrus green mold [1]. Azoxystrobin (strobilurin) was registered as a new fungicide in the USA for the control of postharvest diseases of citrus [67,68]. However, due to its site-specific mode of action, as mentioned above, it has a high risk of developing resistance in target phytopathogenic fungal populations. Pd isolates collected from various packaging in China were shown to be highly sensitive to azoxystrobin even though it had never previously been used for the control of citrus diseases, indicating the lack of resistant biotypes in the natural population [69]. Although Pd has a high potential to develop resistance to azoxystrobin, no resistance has been described naturally so far. Only a moderate level of resistance to strobilurins were found in some of the Pd isolates evaluated, which shows that strobilurins are effective [35].

The main mechanism of resistance to QoI is based on the target site and involves changes in the mitochondrial cytochrome b (CYTB) gene, resulting in variations in the peptide sequence that prevent fungicide binding. Mutations affecting sensitivity to QoI fungicides have been identified in two areas of CYTB, which are related to amino acid positions 120–155 and 255–280 of the encoded protein. This mechanism that underlies resistance to azoxystrobin has been reported in several important phytopathogenic fungi [70,71,72,73,74,75]. In most cases where resistance to strobilurins has been described, resistance was conferred by substitution of a single amino acid (alanine for glycine) at code 143 (G143A) in the cyt b gene. Furthermore, substitution in code 129 for leucine by phenylalanine (F129L) was also found to confer resistance to QoI in some species of fungi, although the level of resistance was lower than that conferred by the G143A substitution [14,76]. Recently, an additional amino acid substitution from glycine to arginine at position 137 (G137R) was also associated with resistance to QoI [77]. In Pd, only UV-induced azoxystrobin-resistant mutants were found. These Pd mutants were genetically stable, and their high levels of azoxystrobin resistance were conferred by a single point mutation (G143A) in the Pdcyt b gene [69].

The second mechanism of resistance to QoI fungicides is mediated by the induction of alternative cyanide-resistant respiration sustained by alternative oxidase (AOX) [78]. In this rescue mechanism, mitochondrial electron transfer is deviated, bypassing the QoI inhibitory site in the cytochrome bc1 complex. Under field conditions, alternative respiration appears to have limited impact on the protective activities of QoI fungicides [79].

### 3.4. Succinate Dehydrogenase Inhibitors (SDHIs)

The target of boscalid is succinate dehydrogenase (SDH) in the mitochondrial electron transport chain. The SDH enzyme catalyzes the oxidation of succinate to fumarate in the mitochondrial matrix, coupling with the decrease in ubiquinone to ubiquinol in the membrane during aerobic respiration [80,81]. SDHI fungicides specifically inhibit fungal respiration by preventing ubiquinone binding sites in the mitochondrial complex II [81].

SDHIs, such as boscalid, fluxapyroxad, penthiopyrad, isopyrazam, and fluopyram, have a spectrum of activity against a wide variety of fungal pathogens in different crops.

Boscalid is a succinate dehydrogenase inhibitor (SDHI) fungicide that is very effective in preventing a large number of plant pathogens, including *Sclerotinia sclerotiorum*, *Botrytis cinerea*, *Alternaria alternata*, and *Corynespora cassiicola* [82,83,84,85]. An amino acid modification in the highly conserved subunit SDH-B is directly related to the binding of SDHI to the target and has been described in different plant pathogens. In SDHI-resistant isolates, histidine at orthologous positions 277, 272, and 267 were substituted in *A. alternata* (B-H277Y/R) [86], *B. cinerea* (B-H272Y/R/L) [87], and laboratory mutants of *Z. tritici* (B-H267Y/L/F/N/Q) [88,89]. Recently, consecutive treatments over several generations with boscalid in the laboratory were shown to result in resistance in Pd. Studies showed that boscalid inhibited SOD activity while POD activity increased, which may be the reason for the increased O_2_− and decreased H_2_O_2_ concentrations in Pd [90]. High levels of ROS are harmful and cause oxidative damage to organisms, but they also play an important role in the regulation of a variety of biological functions [91].

Boscalid is a single-site fungicide and is therefore considered to have a high potential for resistance development regardless of its high activity against Pd. The Fungicide Resistance Action Committee [92] classified SDHI fungicides as medium to high risk with respect to the development of resistance (Table 1) based primarily on single-site mutations of the gene encoding the enzyme succinate target dehydrogenase. The reported resistance has been limited to generation I carboxin fungicides as well as generation II SDHI boscalid.

### 3.5. Phenylpyrroles (PPs) and Anilinopyrimidines (Aps)

Fludioxonil and pyrimethanil are included in the classes of phenylpyrrole (PPs) and anilinopyrimidine (APs), respectively. Both fungicides are very effective in preventing the germination of conidia and the elongation of the germ tube of *P. expansum* and *B. cinerea* [93], and both are registered in a large number of crops as postharvest fungicides and incorporated for postharvest use in citrus [94]. While fludioxonil- and pyrimethanil-resistant Pd isolates have been naturally identified in packinghouses, they are not associated with crop diseases [1]. Fludioxonil is used alone or in combination with azoxystrobin in the control of green mold and other postharvest diseases of citrus. In California citrus packinghouses, a reference sensitivity to fludioxonil has been established in Pd populations [1]. Nevertheless, it was not until 2015 that the first incidence of resistance to fludioxonil in Pd collected from commercial citrus packinghouses was reported after the introduction of the fungicide in the market [95]. Pyrimethanil-resistant isolates were also obtained from different orchards [96].

The mode of action and resistance mechanisms for both classes of fungicides have been carried out in mutants induced in the laboratory or in field isolates of various fungi. Although two nucleotide substitutions were found in a sequence analysis of the N-terminal amino acid repeat region of the os-1-related histidine kinase gene among Pd isolates, these were not correlated with fludioxonil resistance. Studies indicate that the mode of action of fludioxonil on Pd is probably the mitogen-activated protein kinase pathway, which stimulates glycerol synthesis in sensitive and resistant strains [1]. In addition, while pyrimethanil is believed to inhibit the biosynthesis of methionine and other amino acids and the secretion of hydrolytic enzymes involved in the infection process in different fungal pathogens [97], methionine biosynthesis is not the main target of APs in Pd [1]. Therefore, the mechanisms of resistance to fludioxonil and pyrimethanil in Pd remain to be elucidated.

## 4. Resistance-Mediated Drug Efflux Transporters

Efflux transporters can allow fungi to survive exposure to toxic compounds, eliminating the accumulation of compounds in toxic concentrations within fungal cells. These membrane-bound proteins are known to provide protection against a wide range of naturally occurring and xenobiotic toxic compounds [98]. Many studies have reported links between enhanced efflux transporter activity and the appearance of resistance in different fungal pathogens [41,99,100,101,102] including Pd, indicating that efflux transporters may have a common and critical role in fungicide sensitivity. In addition, coincident resistance to many chemical types of fungicides was found to be attributable to overexpression of efflux pumps in some important fungal pathogens.

**Figure 4:**
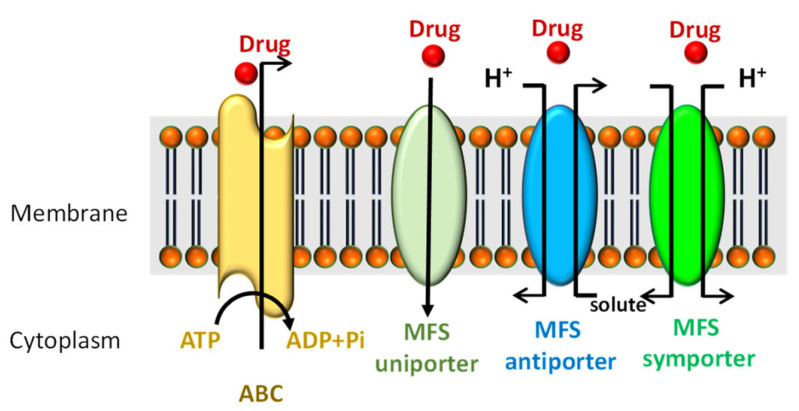
ABC and MFS transporters. ABC: ATP-binding cassette transporter superfamily, MFS: major facilitator superfamily.

Drug efflux transporters are integral membrane-bound proteins that transport an extensive variety of compounds, such as protein macromolecules, ions, or small molecules in a biological membrane [103]. Two major groups of drug transporters have been characterized in fungi, including ABC (ATP-binding cassette) transporters and MFS (major facilitator superfamily) transporters. Multidrug and toxic compound extrusion (MATE), another type of transporter that has been mainly reported in bacteria [104], is related to resistance to antimicrobial agents and was recently reported to be involved in prochoraz resistance in Pd in trancriptomic analysis [105]. In this section, the general function of drug efflux transporters related to resistance to fungicides in the Pd–citrus pathosystem are reviewed (Figure 4).

### 4.1. ATP-Binding Cassette Transporters (ABC)

ATP-binding cassette transporters (ABC) make up one of the largest protein families described to date. The family of ABC transporters is one of the most relevant efflux pumps that exert protection of fungi against chemical compounds [106,107]. These transporters constitute primary active transport systems as they obtain the energy required for transport owing to the hydrolysis of ATP (Figure 4). In filamentous fungi, ABC transporters can act against synthetic fungicides or compounds produced by competing microorganisms [108]. The phenomenon, described as the simultaneous resistance to several chemically unrelated compounds (MDR), is related to the overexpression of ABC transporters due to the resulting pleiotropic effects. Four ABC transporters have been identified in Pd: PMR1, PMR3, PMR4, and PMR5. Of them, only PMR1 [48,109] and PMR5 [110] appear to be related to multidrug resistance in Pd. A more exhaustive characterization of the four transporters showed that while no genetic changes were detected between isolates in PMR1, PMR3, and PMR4, some specific modifications were observed in the promoter and coding regions of PMR5 in strains resistant to both TBZ and different DMI fungicides [35]. Furthermore, the presence of toxic substances selectively activates the expression of PMR1 and PMR5. Specifically, triflumizole and imazalil activate PMR1 transcription, while benzimidazoles, dithianone, and resveratrol promote PMR5 transcription. Thus, Pd resistance can be determined by selective transcriptional activation of ABC transporter genes to a toxic compound. [110]. Moreover, an exhaustive search of putative ABC genes in *Pd* identified a total of 46 chromosome-encoded ABC family transporters. Analysis of these genes revealed that five more ABC transporters may be involved in drug resistance as they were upregulated in imazalil-inducing expression analysis [64]. Furthermore, transcriptome analysis of prochloraz-treated Pd strains revealed three new ABC transporters that were more involved in prochloraz resistance [111].

### 4.2. Major Facilitator Superfamily Transporters (MFS)

MFS transporters are part of the family of active secondary transporters that can transport substances in response to ionic gradients. MFS transporters can mediate antiport, uniport, and symport of different products [112] (Figure 4). MFS transporters can serve as drug transporters owing to a proton gradient, which allows it to confer multidrug resistance (MDR). Many of these MFS transporters transport small molecules in response to ionic gradients in such a way that they function as an H + antiporter in microorganisms, regulating their growth under stress conditions as they affect the membrane potential and internal pH [113].

These transporters could play a role in sensitivity to different compounds as they usually have a narrow substrate affinity that guarantees an important contribution in the transaction of a wide range of substrates. The effect on toxin efflux and fungicide sensitivity has been observed in many fungal MFS transporters. For instance, suppression of the *Cercospora nicotianae* MFS transporter reduced the cercosporin toxin [114]. In *B. cinerea*, *BcMfs1* affected sensitivity to camptothecin and cercosporin and resistance to DMI [115] and *mfsM2* showed greater efflux fungicidal activity [99]. The elimination of *MgMfs1* from *M. graminicola* contributed to strobilurin fungicide resistance but not other evaluated fungicides [116,117]. In *Zymoseptoria tritici*, the *MgMFS1* transporter participated in the MDR phenotype [110]. Furthermore, the *AaMFS19* MFS transporter was shown to play a role in resistance to oxidative stress and chemicals in the phytopathogenic fungus *Alternaria alternata* [118]. Transcriptome analysis of MDR strains of *P. expansum* reported overexpression of MFS transporter genes before or after exposure to fludioxonil [119].

In Pd, more than 100 MFS have been identified due to the availability of the Pd genome [5]. So far, of all the identified Pd–MFS transporters, seven have been characterized more thoroughly, namely *PdMfs1* [120], *Pdmfs2* [121], *PdMFS1* [101], *PdMFS2, PdMFS3, PdMFS4*, and *PdMFS5* [102]. All are involved in some way in resistance to chemical fungicides and in some cases may contribute to an increase in fungal virulence. An analysis of each of the proteins encoded by these MFS genes shows that they share little homology between them, which also affects their functionality. Thus, while *PdMfs1* has a clear effect against imazalil, *Pdmfs2* and *PdMFS1* play an important role in prochloraz resistance. Both are also involved in processes developed during the fruit–pathogen interaction, such as conidia and the progress of fungal disease. Furthermore, *PdMFS1* is able to confer MDR phenotype as it contributes to the output of a wide range of fungicidal compounds [101]. Among the latest identified Pd–MFS transporters, only *PdMFS2* and *PdMFS3* appear to participate in fungicide resistance. Both genes contribute to simultaneous resistance to several unrelated toxic compounds (MDR phenotype), as previously reported for other fungal MFS transporters [101,113,122]. The phylogenetic analysis of a large number of these MFS transporters in Pd revealed all the genes had different genetic structures and encoded proteins of different sizes and that only PdMFS2p appeared together with the group that comprised the MFS transporters assigned as drug efflux transporters [102]. On the other hand, the transcriptomic analysis carried out in Pd after treatment with prochloraz highlighted overexpression of 14 different MFS transporters [111].

MFS transporters have been linked to QoI resistance. In *MgMfs1*, which encodes an MFS transporter gene from *M. graminicola*, the deletion of *MgMfs1* showed insensitivity to QoI [116]. However, until now, no decrease in sensitivity or resistance to QoI fungicides has been identified in Pd mediated by an MFS transporter. However, the contribution of these energy-dependent mechanisms in adaptation to fungicides by phytopathogenic fungi should be seriously considered despite the scarcity of data on resistance to efflux transporter-based QoI fungicides.

Until now, the contribution of MFS transporters as a decisive factor in the plant–pathogen interaction is unknown [37], and further functional characterization of more different MFS transporters will be necessary to establish their role in the Pd–citrus interaction.

## 5. Regulation of Fungicide Resistance

### 5.1. Transcription Factors in Pd Fungicide Resistance

Transcription factors (TF) are involved in transcriptional regulation and play a relevant role in fungal interactions. TFs can contribute to primary or secondary metabolism [123], along with stress responses and sensitivity to pleiotropic drugs [124].

SREBP transcription factors, which contain a bHLH domain, function as critical controllers of sterol homeostasis and are universally found in fungi. In most fungi, SREBPs play a crucial role in controlling ergosterol biosynthesis [125]. In Pd, the SREBP protein SreA was initially identified and characterized, which plays an important role in prochloraz resistance and in the transcription of ergosterol synthesis genes [111]. Evidence on the transcriptional regulation of these target genes has emerged to explain the drug-resistant mechanisms of Pd. In the citrus postharvest pathogen Pd, there is another SREBP homolog, *PdsreB*, which appears to be involved in fungicide resistance and in the control of *CYP51* gene expression [126]. Functional characterization showed the two genes (*PdsreA* and *PdsreB*) act as global controllers in a great variety of biological functions, especially in aspects that mediate ergosterol biosynthesis and resistance to fungicides. Thus, the expression of the ERG1 gene (in the ergosterol pathway) is regulated by both the *PdsreA* and *PdsreB* genes, while only PdsreA is involved in the expression of ERG2. As both genes regulate different aspects, as has been shown with single and double mutations of the genes, it is possible that there are other transcription factors involved in ergosterol biosynthesis that could be activated when both SREBPs are inhibited [126]. Furthermore, it is possible that the SREBP genes play a relevant role in the control of certain MFS transporters in Pd as some of them were found to be overrepresented in gene transcription studies [126].

Fungi are known to use the transcriptional regulation of genes encoding efflux transporters to detoxify certain compounds. The expression of efflux transporters is controlled mainly by fungal zinc group transcription factors (TF [Zn2Cys6]) [127]. Fungi apparently regulate and manage different stages of the detoxification system by modifications in particular transcription factors, and this regulatory system seems to be conserved in filamentous fungi [128]. Modifications in the activity of transcriptional regulators elicit overproduction of MFS transporters [129].

Transcriptional regulator Ste12 might function as a regulator of pathway-specific genes [130]. In Pd, *PdSte12* might be involved in the control of expression of several genes through repression or activation, triggering multiple responses, such as detoxification. *PdSte12* acts as a negative regulator in several genes involved in transport, including the primary ABC transporters (PMR1 and PMR5) and the secondary MFS transporters (*PdMFS1-6*). *PdSte12* also positively controls sterol demethylases (*CYP51* and *PdCYP51B*) [131].

**Figure 5:**
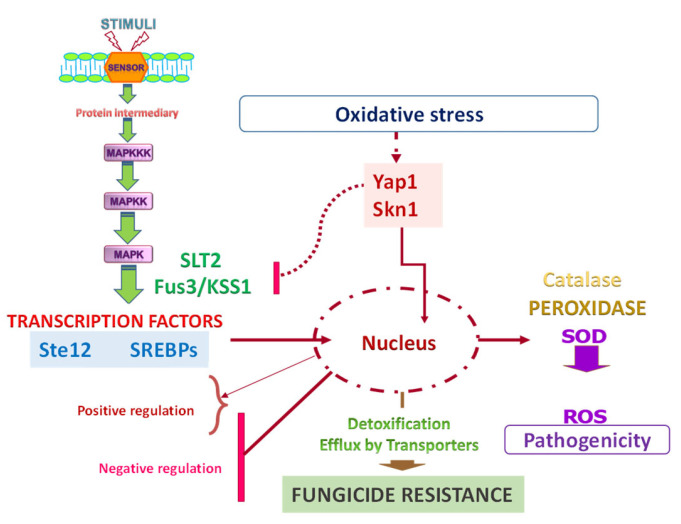
Schematic of regulatory mechanisms involved in fungal resistance that are also related to fungicide virulence and oxidative stress.

*Skn7* is a highly conserved stress-responsive transcription factor and, apart from *Ssk1/SskA*, the second response regulator that can be activated via the phosphotransfer protein Ypd1. *Skn7* plays a well-established role in the oxidative stress response. *Skn7* is involved in maintenance of the cell wall integrity of *S. cerevisiae* and other fungi. Although these genes have not been identified to date in Pd, in the MFS transporters of *A. alternata* (*AaMFS19* and *AaMFS54*), gene expression is simultaneously regulated by the stress-sensitive transcription factor Yap1. The expression of *AaMFS19* is also controlled by the stress-related regulator *Skn7* [118,132], but this regulator does not affect *AaMFS54*. ROS resistance in *A. alternata* is, at least in part, mediated by membrane-bound transporters as regulators *Yap1* and *Skn7* have been shown to play a critical role in resistance to oxidative stress [133] (Figure 5).

### 5.2. Protein Kinases in Pd Fungicide Resistance

Fungi processing is controlled by protein kinase (PK) cascades [134]. MAPKs are involved in signaling pathways that are highly conserved in all eukaryotic organisms. There are three orthologous MAPKs in filamentous fungi, namely Hog1, Slt2, and Fus3/Kss1 [135]. The MAPK Hog1, Slt2, and Fus3/Kss1 orthologous in Pd, called *Pdos2*, *PdSlt2*, and *PdMpkB*, have been identified and characterized [46,136,137,138]. Hog1-like MAPKs, which are highly conserved among various fungi, possess different physiological functions, including a high osmolarity adaptation [139]. *Pdos2* is involved in osmotic adaptation and is associated with positive control of glycerol synthesis and negative regulation of ergosterol synthesis [138]. The mode of action of fludioxonil on Pd is probably via the mitogen-activated protein kinase pathway, which promotes glycerol synthesis [1].

*PdSlt2* functions as a negative regulator of different genes involved in transport, comprising primary transporters (ABC transporters PMR1 and PMR5) and secondary transporters (MFS transporters *PdMFS1-6*). In contrast, *PdSlt2* positively controls sterol demethylases *PdCYP51A* and *PdCYP51B* [137]. In this sense, the control of important genes involved in fungicide resistance highlights the role of this MAPK in mediating the process involved in resistance to fungicides.

Figure 5 illustrates how transcriptional regulation plays an important role in fungal interactions and how signal transduction pathways can be interconnected. Furthermore, oxidative stress and the ROS response may also be part of fungal–plant interaction as they are simultaneously involved in fungal pathogenicity and resistance to fungicides.

## 6. Conclusions

The mechanisms responsible for acquiring resistance to fungicides in Pd are due to numerous genes that in some cases depend on the type of fungicide. Resistance to a certain fungicide can be defined by modifications in a single gene. However, in general, different mechanisms, such as variations that modify the binding target of the fungicide or changes in gene expression, determine the appearance of resistance to toxic compounds.

**Figure 6:**
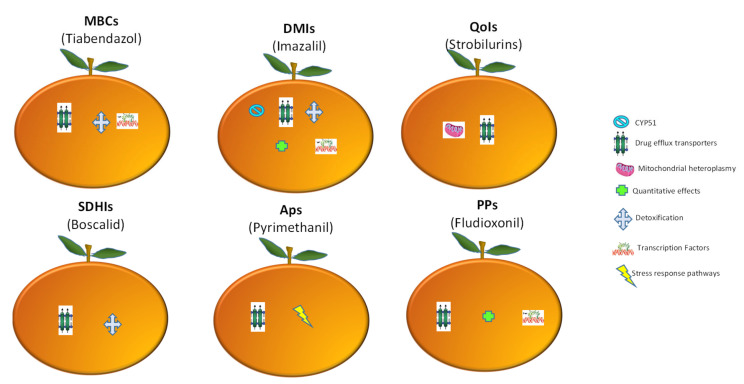
Mechanisms of resistance to main types of fungicides used for green mold disease management. Each orange represents a chemical class, whereas the cryptograms within the oranges correspond to a resistance process as shown in the figure. MBCs: methyl benzimidazole carbamates, DMIs: demethylation inhibitors; QoIs: quinone outside inhibitors; SDHIs: succinate dehydrogenase inhibitors; APs: anilinopyrimidines; PPs: phenylpyrroles. Adapted from Hu and Chen [105].

The complexity of genomic pathways in Pd populations has allowed them to respond and adapt to fungicides in multiple ways. At least seven mechanisms that can cause resistance to various classes of chemical fungicides in Pd have been described (Figure 6). Remarkably, resistance caused by increased activity of drug efflux pumps has been the most notorious for all major chemical classes, indicating its common role in resistance evolution. Other mechanisms mentioned above, such as detoxification and transcription factors, have also been found to be relevant.

Fungicides play a determining role in the control of crop pathogens, and it is likely that they will continue to be one of the most relevant means in the future to prevent the development of diseases. An approach that integrates plant breeding and biotechnology, the development of chemical compounds, and policies that ensure the use of fungicides in a sustainable way through innovation in alternative technologies is essential to achieve the challenge of food safety in a changing environment and counteract risks in plant health and postharvest citrus fruits in particular.

Expanding knowledge of fungal resistance mechanisms not only allows the design of faster molecular tools to rapidly detect fungal resistance but can also allow the identification of natural secondary metabolites and the design of new antifungal compounds that are more efficient and specific.

## Figures and Tables

**Table 1:** Types of fungicides used in citrus control programs and their targets.

FRAC Code	Fungicide Class	Celular Function Affected	Target Protein	Risk Resistance Development
1	Methyl benzimidazoles (MBCs)	Cytoskeleton	β-tubulin	High
3	Demethylation inhibitors (DMIs)	Membrane biosynthesis	Sterol 14α-demethylase (CYP51)	Medium
11	Quinone outside inhibitors (QoIs)	Respiration	Mitochondrial cytochrome b	High
7	Succinate dehydrogenase inhibitors (SDHIs)	Respiration	Succinate dehydrogenase	Medium to High
12	Phenylpyrroles (PPs)	Altered target site (protein kinase involved in osmoregulation)	Protein kinase	Low to medium
9	Anilino-pyrimidines (APs)	Altered target site (protein kinase involved in osmoregulation)	Protein kinase	Medium
